# Is Respiratory Viral Infection an Inciting Event in the Development of Melioidosis? A Systematic Evaluation of Co-infection With *Burkholderia pseudomallei* and SARS-CoV-2 or Influenza

**DOI:** 10.1093/ofid/ofae700

**Published:** 2024-12-04

**Authors:** Genevieve E Martin, Jerry L J Chen, Celeste Woerle, Alexandra Hinchcliff, Robert W Baird, Jane Davies, Bart J Currie

**Affiliations:** Global and Tropical Health Division, Menzies School of Health Research, Charles Darwin University, Darwin, Australia; Department of Infectious Diseases, Royal Darwin Hospital, Darwin, Australia; Department of Infectious Diseases, The University of Melbourne at the Peter Doherty Institute for Infection and Immunity, Melbourne, Australia; Centre for Disease Control, Public Health Division, NT Health, Darwin, Australia; National Centre for Epidemiology and Population Health, Australian National University, Canberra, Australia; Global and Tropical Health Division, Menzies School of Health Research, Charles Darwin University, Darwin, Australia; Global and Tropical Health Division, Menzies School of Health Research, Charles Darwin University, Darwin, Australia; Department of Infectious Diseases, Royal Darwin Hospital, Darwin, Australia; Territory Pathology, Royal Darwin Hospital, Darwin, Australia; Global and Tropical Health Division, Menzies School of Health Research, Charles Darwin University, Darwin, Australia; Department of Infectious Diseases, Royal Darwin Hospital, Darwin, Australia; Global and Tropical Health Division, Menzies School of Health Research, Charles Darwin University, Darwin, Australia; Department of Infectious Diseases, Royal Darwin Hospital, Darwin, Australia

**Keywords:** melioidosis, SARS-CoV-2, influenza

## Abstract

Respiratory viral infection may increase infection with *Burkholderia pseudomallei* progressing to clinical disease (melioidosis). This data linkage study evaluated associations between melioidosis and SARS-CoV-2 or influenza. Among 160 melioidosis cases, there was no difference in risk factors, vaccine status, or disease severity between 17 with viral co-infection and 143 without.

## Graphical Abstract

**Graphical abstract:**
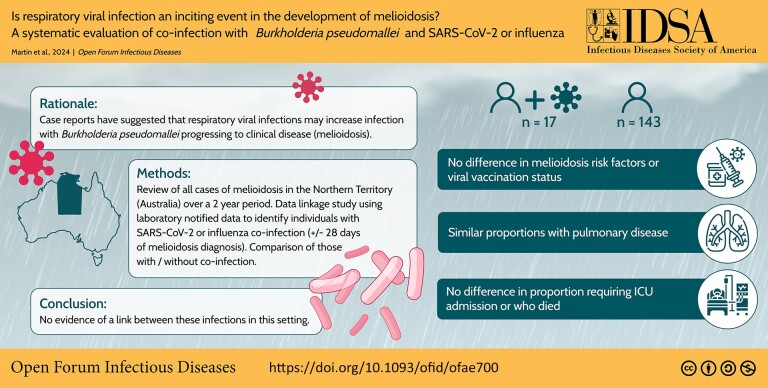
This graphical abstract is also available at Tidbit: https://tidbitapp.io/tidbits/is-respiratory-viral-infection-an-inciting-event-in-the-development-of-melioidosis-a-systematic-evaluation-of-co-infection-with-burkholderia-pseudomallei-and-sars-cov-2-or-influenza

## Teaser

This systematic evaluation of co-infection with SARS-CoV-2 or influenza and Burkholderia pseudomallei (causing melioidosis) assessed a possible link between these infections - but found no difference in risk factors, disease severity or outcomes between those with and without viral co-infection.

Infection with *Burkholderia pseudomallei* occurs in tropical and subtropical regions, with subsequent clinical disease (melioidosis) causing substantial morbidity and mortality [1]. Melioidosis classically occurs in individuals with risk factors including diabetes mellitus, hazardous alcohol consumption, chronic renal and lung disease, and immunosuppression [2, 3]. The presentation of melioidosis is varied, with pulmonary involvement seen in approximately half of cases [2, 3].

Two instances of melioidosis have been reported in nonendemic areas following hospital admission with influenza A [4] or SARS-CoV-2 [5] infection. In both cases, historical epidemiological exposure to *B pseudomallei* had occurred (6 and at least 1 year prior), and there was a clear temporal relationship between respiratory viral infection and development of melioidosis [4, 5]. Other cases of co-infection with melioidosis and SARS-CoV-2 have been reported [6–8], including an outbreak at a COVID-19 field hospital [9], supporting the possibility of viral infection as a triggering event for the activation of latent *B pseudomallei* and for progression of clinical disease after recent infection.

Both SARS-CoV-2 and influenza can lead to the development of significant co-infections. For example, risk of invasive infection with *Aspergillus* (and *Mucorales*) species is increased following infection with both SARS-CoV-2 and influenza [10–12] in which fungal infection risk occurs through viral modulation of immune responses [13]. The reactivation of other latent infections including tuberculosis [14] and herpesvirus infections [15] have also been associated with both respiratory infections, and secondary bacterial pneumonia is a well-recognized postviral sequela [16]. It is therefore plausible that SARS-CoV-2 or influenza could similarly increase the risk of melioidosis. There have, however, been no systematic assessments of whether SARS-CoV-2 or influenza increases the risk of melioidosis or of the impact of co-infection on disease severity.

In this study, we aimed to systematically identify cases of melioidosis with co-infection of SARS-CoV-2 or influenza in the Northern Territory (NT) of Australia, a region where melioidosis is endemic [3], and to describe the characteristics and disease outcomes of these cases.

## METHODS

### Case Identification

Laboratory diagnoses of melioidosis, influenza, and SARS-CoV-2 are all notifiable to NT public health authorities. Cases of all 3 infections were identified from 10 January 2021 through 30 September 2023, aligning with the start of the 2021 melioidosis season (which occurs with the monsoonal wet season [3]) and the first cases of community transmission of SARS-CoV-2 in the NT [17]. Cases of influenza and melioidosis were sourced from the NT Notifiable Disease System. SARS-CoV-2 data (including cases testing positive by rapid antigen test until 10 November 2022 and polymerase chain reaction throughout the study period) were sourced from an internet-based data storage system (REDCap). Respiratory virus testing was performed as part of routine clinical care; local testing policies varied during the study period and broadly included testing of all individuals presenting with febrile illnesses and those admitted to hospital. Most, but not all, cases will therefore have received a respiratory viral test around the time of diagnosis.

Deterministic linkage was performed to identify individuals with melioidosis and influenza or SARS-CoV-2 co-infection (defined as diagnosis within 28 days either side of melioidosis diagnosis) using a unique identification number within the NT healthcare system, then given name, surname, and date of birth. Manual review was performed of additional cases where the Darwin Prospective Melioidosis Study (DPMS) [3] record contained the keyword “COVID.”

### Clinical Data

Clinical data and outcomes of melioidosis were collected from the DPMS. Dates of influenza and SARS-CoV-2 vaccination were extracted from the Australian Immunisation Register for all individuals. Daily rainfall data for Royal Darwin Hospital site were sourced from the Bureau of Meteorology.

### Statistical Analysis

Data were analyzed using R (v4.3.2, packages tidyverse [v2.0.0] and zoo [v1.8-12]). Differences between groups were assessed using Wilcoxon rank-sum test, Pearson χ^2^ test, or Fisher exact test as appropriate. For all tests, *P* values <.05 were considered statistically significant.

### Ethics Approval

Recruitment into the DPMS is approved by the Human Research Ethics Committee of the Northern Territory Department of Health and Menzies School of Health Research (approval number 02/38).

## RESULTS

**Figure 1.**
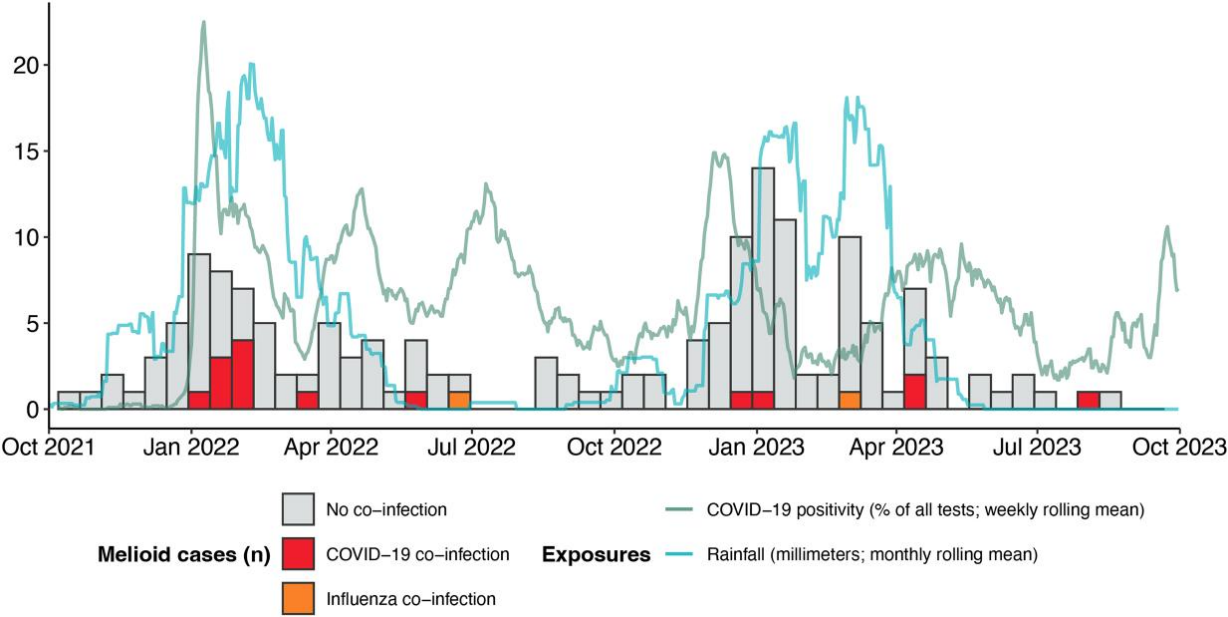
Timing of diagnoses of melioidosis and COVID-19 or influenza over study period. Histogram shows the number of cases of melioidosis with blocks coloured by the presence of COVID-19 (red) or influenza (orange) co-infection, or an absence of co-infection (gray). Local weekly rolling mean of COVID-19 test positivity is shown as a green line (expressed as a percentage of all tests from Darwin performed by Royal Darwin Hospital pathology service). Local rainfall (expressed as a monthly rolling mean in milliliters) is shown as a blue line.

A total of 160 episodes of culture-confirmed melioidosis from 158 individuals were diagnosed; 17 were linked to either influenza (2 cases) or SARS-CoV-2 (15 cases). Manual review was performed of 7 additional cases, none of which met the criteria for inclusion. Of those with a co-infection, the respiratory virus was diagnosed within the week prior or same day as melioidosis for 9 individuals (53%; Supplementary Figure 1). Most SARS-CoV-2 cases (9 individuals) occurred between January and March 2022, coinciding with a major peak in local COVID-19 test positivity (Figure 1). The number of cases of melioidosis peaked twice over the study period, concurrent with peak rainfall.

Individuals with and without respiratory viral co-infection were similar with regards to sex, age, ethnicity, and risk factors for melioidosis (Table 1), which mirror those previously reported in the DPMS [3]. Most individuals had a risk factor for developing melioidosis, with diabetes being the most common in both groups (71% in co-infected vs 53% with melioidosis alone, *P* = .2).

**Table 1.** Characteristics of Individuals With Melioidosis and with or Without COVID-19 or Influenza Co-Infection

Respiratory Viral Co-Infection	N	No^a^ N = 143	Yes n = 17	*P* Value
*Patient-level characteristics* ^b^	158	…	…	
Sex				.3
Female	…	62 (44%)	5 (29%)	
Male	…	79 (56%)	12 (71%)	
Age (y)	…	52 [43–62]	52 [42–66]	.8
Ethnicity				
Aboriginal and/or Torres Strait Islander	…	86 (61%)	11 (65%)	.8
Other	…	55 (39%)	6 (35%)	
Melioidosis risk factors				
Diabetes	…	75 (53%)	12 (71%)	.2
Hazardous alcohol use	…	45 (32%)	5 (29%)	.8
Chronic renal disease	…	17 (12%)	3 (18%)	.5
Chronic lung disease	…	40 (28%)	4 (24%)	.8
Malignancy	…	20 (14%)	1 (5.9%)	.5
Immunosuppressive therapy	…	12 (8.5%)	1 (5.9%)	>.9
No clinical risk factor identified	…	25 (18%)	1 (5.9%)	.3
*Episode-level characteristics*				
Episode type	160	…	…	.3
Acute	…	123 (86%)	16 (94%)	
Chronic	…	13 (9.1%)	0	
(Re)activation from latency	…	1 (0.7%)	1 (5.9%)	
Reinfection	…	3 (2.1%)	0	
Relapse	…	3 (2.1%)	0	
Bacteremia	160	75 (52%)	10 (59%)	.6
Pneumonia as primary site of infection	160	78 (55%)	11 (65%)	.4
Neutrophils (cells × 10^9^/L)	91	8.8 [5.7–12.4]	7.4 [5.9–16.3]	.9
Lymphocytes (cells × 10^9^/L)	90	1.3 [0.60–2.05]	1.2 [0.80–2.20]	>.9
C-reactive protein (mg/L)	87	141 [50–233]	177 [55–301]	.6
HbA1c (%)	83	6.7 [5.8–11.3]	8.7 [6.4–10.9]	.6
Disease severity				
Septic shock	160	32 (22%)	0	.025
Admitted to ICU	160	31 (22%)	1 (5.9%)	.2
Died	160	…	…	.13
Yes, related to infection	…	13 (9.1%)	0	
Yes, unrelated to infection	…	6 (4.2%)	2 (12%)	

Numbers are shown as n (%) for categorical variables and median (interquartile range) for continuous variables. Groups have been compared with Pearson chi-squared test or Fisher exact test (categorical) or Wilcoxon rank-sum test (continuous variables).

Abbreviation: ICU, intensive care unit.

^a^Includes 2 individuals who had 2 separate treatment episodes over the study period.

^b^Shown for diagnosis of first episode.

Disease characteristics and indicators of severity did not differ between those with and without respiratory viral co-infection, with similar proportions experiencing bacteremia (*P* = .6) and having pneumonia as the primary focus of infection (*P* = .4). The majority of disease episodes represented acute infection (94% in co-infected vs 84% with melioidosis alone, *P* = .3), with only a single case in the co-infection group (influenza) favored to represent activation from latency [18]. Only 1 melioidosis patient with respiratory viral co-infection was admitted to the intensive care unit and the 2 deaths in those with co-infection were attributed to other causes (acute stroke and metastatic malignancy) and not either infection (Table 1).

Antiviral therapeutics were provided to most of those with a respiratory viral co-infection (100% with influenza and 67% with SARS-CoV-2; Supplementary Table 1). Of those for whom vaccine data were available, most had received at least 1 dose of SARS-CoV-2 vaccine before the development of melioidosis (100% in SARS-CoV-2 co-infected vs 93% without SARS-CoV-2, *P* = .6), with no difference in the number of doses received or time since most recent dose (Supplementary Table 2). Neither of the 2 individuals with influenza co-infection, and only 45% of those without, had received an influenza vaccine in the 12 months prior.

## DISCUSSION

Here we report infection with SARS-CoV-2 or influenza within 28 days of a diagnosis of melioidosis in 17 individuals, comprising 11% of melioidosis cases in the NT over the study period. Our data represent the first systematic assessment of co-occurrence of respiratory viral infections and melioidosis.

The most compelling data about a causative link thus far comes from case reports from nonendemic areas where *B pseudomallei* exposure was historic to disease development and melioidosis occurred shortly after respiratory virus infection [4, 5]. These cases represented activation of *B pseudomallei* from latency estimated to represent <3% of all melioidosis cases [18]. A COVID-19 field hospital outbreak of melioidosis in Thailand linked to tap water contaminated with *B pseudomallei* may have provided evidence of concurrent viral co-infection driving development of melioidosis in those exposed to *B pseudomallei* [9]. Furthermore, the fatal case of a 5-year-old who died from disseminated melioidosis with concurrent COVID-19 infection [7] suggests COVID-19 may adversely impact the course and severity of melioidosis.

In our study, however, the risk factor profiles of those who developed melioidosis were similar between those with and without respiratory viral co-infection. Despite plausible hypotheses of a pathophysiological link [7, 9, 18], our results are not supportive of respiratory viral co-infection representing a major risk factor for the development of melioidosis in our setting.

It is important to note that our and others' cases of melioidosis and SARS-CoV-2 co-infection [6–9] all occurred during periods of high SARS-CoV-2 transmission and testing. In our study, most cases co-infected with SARS-CoV-2 and melioidosis were diagnosed during a peak of local SARS-CoV-2 transmission and at the time of peak rainfall (the major environmental risk factor for melioidosis). The co-occurrence of these risks for transmission of virus and bacterium could well explain the co-infection observed. The apparent peak of diagnosis of respiratory viral infection prior to that of melioidosis in our data could simply reflect diagnostic lag (with testing results for SARS-CoV-2 and influenza taking hours, and culture results for melioidosis taking days).

Of the 28 previously reported cases of SARS-CoV-2 and melioidosis co-infection where disease outcome was described, 40% were fatal [5, 7–9]. Although these cases suggest potential for more severe disease when co-infection occurs, we did not observe poorer outcomes (death or intensive care admission) in those with co-infections. Mortality from melioidosis in our region is lower than in most other endemic settings and access to antibiotics, antiviral therapies, COVID-19 vaccines, and high-quality intensive care facilities may account for our findings [3, 19].

The major strength of this study is the systematic approach needed to avert risks of bias from case reports. We acknowledge, however, that limited numbers of co-infected cases make a type II error (failure to observe a true association between these infections) possible.

## CONCLUSION

This systematic evaluation of co-occurrence of melioidosis and influenza or SARS-CoV-2 infection in tropical Australia did not find evidence to support a previously hypothesized link between the development of these infections. Nevertheless, such a link may still be important in other settings where therapeutic resources differ, and we encourage further prospective studies.

## Supplementary Material

ofae700_Supplementary_Data

## Supplementary Data

Supplementary materials are available at *Open Forum Infectious Diseases* online. Consisting of data provided by the authors to benefit the reader, the posted materials are not copyedited and are the sole responsibility of the authors, so questions or comments should be addressed to the corresponding author.

## Notes

***Acknowledgments.*** We acknowledge all colleagues who were involved in the clinical management of these individuals and the Royal Darwin Hospital microbiology laboratory for their expertise in diagnosis of melioidosis and for respiratory virus testing. This work was supported by a National Health and Medical Research Council (NHMRC) Medical Research Future Fund (MRFF) Investigator Grant [grant number GNT1194615] to J.D. Funders played no role in the study design, analysis, or the decision to publish.

***Author contributions.*** Study was conceived by B.C. and J.D. Data collection was performed by C.W., A.H., and G.E.M. C.W. was responsible for data management. Diagnostic testing for melioidosis and respiratory viruses was overseen by R.W.B. Data analysis was performed by G.E.M. and J.C. Writing of initial manuscript was performed by G.E.M. Input into interpretation of results was provided by B.C., J.D., and G.E.M. All authors have reviewed and approved the final version.
