# Supplementary material for: Is Respiratory Viral Infection an Inciting Event in the Development of Melioidosis? A Systematic Evaluation of Co-infection With Burkholderia pseudomallei and SARS-CoV-2 or Influenza
Source: Open Forum Infect Dis. 2024 Dec 4;11(12):ofae700. doi: 10.1093/ofid/ofae700 (PMC11632372; doi:10.1093/ofid/ofae700)
Supplement: ofae700_Supplementary_Data [file ofae700_supplementary_data.docx]

**Is respiratory viral infection an inciting event in the development of melioidosis? a systematic evaluation of co-infection with *Burkholderia pseudomallei* and SARS-CoV-2 or influenza**

Genevieve E Martin, Jerry L J Chen, Celeste Woerle, Alexandra Hinchcliff, Robert W Baird, Jane Davies, Bart J Currie

**SUPPLEMENTARY APPENDIX**

**Contents**

[Supplementary Table 1. Antiviral therapeutics provided to individuals with SARS-CoV-2 or influenza coinfection 2](#_Toc180393492)

[Supplementary Table 2. COVID-19 vaccination status of individuals with melioidosis and with or without COVID-19 co-infection 2](#_Toc180393493)

[Supplementary Figure 1. Interval between diagnosis of melioidosis and diagnosis of COVID-19 or influenza co-infection 3](#_Toc180393494)

# Supplementary Table 1. Antiviral therapeutics provided to individuals with SARS-CoV-2 or influenza coinfection

| **Antiviral provided** | **Influenza**  n = 2 | **SARS-CoV-2**  n = 15 |
| --- | --- | --- |
| oseltamivir | 2 (100%) | - |
| nirmatrelvir/ritonavir |  | 2 |
| budesonide |  | 2 |
| nirmatrelvir/ritonavir & budesonide |  | 1 |
| dexamethasone & baricitinib |  | 1 |
| dexamethasone, baricitinib & remdesivir |  | 1 |
| remdesivir, budesonide & sotrovimab |  | 1 |
| prednisolone & remdesivir |  | 1 |
| remdesivir |  | 1 |
| No antiviral therapy provided | 0 (0%) | 5 (33%) |

# Supplementary Table 2. COVID-19 vaccination status of individuals with melioidosis and with or without COVID-19 co-infection

| **COVID-19 co-infection** | **n** | **No**  n = 145 | **Yes**  n = 15 | **p-value** |
| --- | --- | --- | --- | --- |
| Any COVID-19 vaccine doses received prior to infection | 137 | 113 (93%) | 15 (100%) | 0.6 |
| Number of COVID-19 vaccine doses received prior to infection | 137 |  |  | 0.6 |
| - 0 |  | 9 (7.4%) | 0 |  |
| - 1 |  | 6 (4.9%) | 2 (13%) |  |
| - 2 |  | 35 (29%) | 6 (40%) |  |
| - 3 |  | 53 (43%) | 5 (33%) |  |
| - 4 |  | 17 (14%) | 2 (13%) |  |
| - 5 |  | 2 (1.6%) | 0 |  |
| COVID-19 vaccine dose in 6 months prior to infection | 137 | 56 (46%) | 10 (67%) | 0.13 |

Numbers are shown as n (%) for categorical variables and median (interquartile range) for continuous variables. Groups have been compared with Pearson’s chi-squared test or Fisher’s exact test (categorical).

# Supplementary Figure 1. Interval between diagnosis of melioidosis and diagnosis of COVID-19 or influenza co-infection


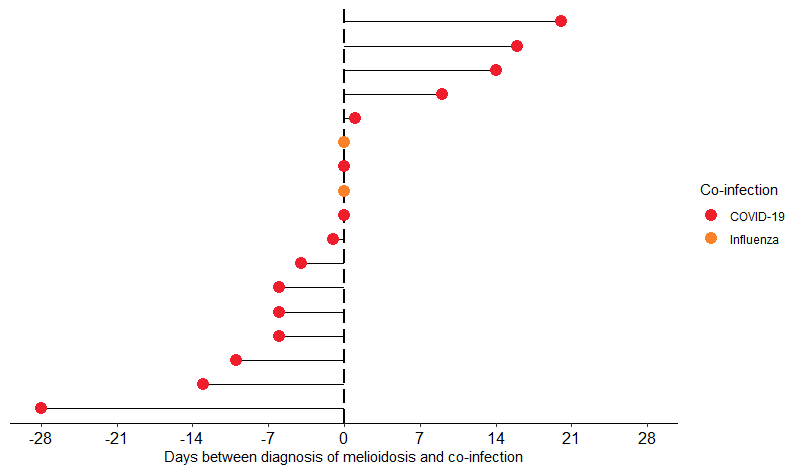


Interval between diagnosis of melioidosis and diagnosis of COVID-19 or influenza co-infection for n=17 individuals infected with both. Interval is shown relative to melioidosis diagnosis (shown at 0), and individuals are ordered by interval.
